# Morphological evidence for telocytes as stromal cells supporting satellite cell activation in eccentric contraction-induced skeletal muscle injury

**DOI:** 10.1038/s41598-019-51078-z

**Published:** 2019-10-10

**Authors:** Mirko Manetti, Alessia Tani, Irene Rosa, Flaminia Chellini, Roberta Squecco, Eglantina Idrizaj, Sandra Zecchi-Orlandini, Lidia Ibba-Manneschi, Chiara Sassoli

**Affiliations:** 1 Department of Experimental and Clinical Medicine, Section of Anatomy and Histology, University of Florence, Florence, Italy; 2 Department of Experimental and Clinical Medicine, Section of Physiological Sciences, University of Florence, Florence, Italy

**Keywords:** Skeletal muscle, Cells

## Abstract

Although telocytes (TCs) have been proposed to play a “nursing” role in resident satellite cell (SC)-mediated skeletal muscle regeneration, currently there is no evidence of TC-SC morpho-functional interaction following tissue injury. Hence, we explored the presence of TCs and their relationship with SCs in an *ex vivo* model of eccentric contraction (EC)-induced muscle damage. EC-injured muscles showed structural/ultrastructural alterations and changes in electrophysiological sarcolemnic properties. TCs were identified in control and EC-injured muscles by either confocal immunofluorescence (*i.e*. CD34^+^CD31^−^ TCs) or transmission electron microscopy (TEM). In EC-injured muscles, an extended interstitial network of CD34^+^ TCs/telopodes was detected around activated SCs displaying Pax7^+^ and MyoD^+^ nuclei. TEM revealed that TCs invaded the SC niche passing with their telopodes through a fragmented basal lamina and contacting the underlying activated SCs. TC-SC interaction after injury was confirmed *in vitro* by culturing single endomysial sheath-covered myofibers and sprouting TCs and SCs. EC-damaged muscle-derived TCs showed increased expression of the recognized pro-myogenic vascular endothelial growth factor-A, and SCs from the same samples exhibited increased MyoD expression and greater tendency to fuse into myotubes. Here, we provide the essential groundwork for further investigation of TC-SC interactions in the setting of skeletal muscle injury and regenerative medicine.

## Introduction

Telocytes (TCs) are unique stromal cells newly identified in a variety of organs over the last decade^1^. These peculiar interstitial cells are ultrastructurally characterized by a small nucleated cell body with a scarce cytoplasm that abruptly gives rise to a variable number of extremely long and thin processes, called telopodes, which often exhibit a sinuous trajectory and a dichotomous branching pattern. Telopodes represent the most distinctive feature of TCs and are morphologically recognizable because of their moniliform appearance conferred by the irregular alternation of slender segments, termed podomers, and small cistern-like dilations, referred to as podoms, which accommodate few organelles such as mitochondria, endoplasmic reticulum cisternae and caveolae^2,3^. The morphology of the cell body varies depending on the number of telopodes, being typically piriform for one, spindle-shaped for two, triangular for three and stellate for more processes^3^. Besides the above described ultrastructural traits, it appears that TCs differ from other stromal cells, such as ‘classical’ fibroblasts/fibrocytes and mesenchymal stem cells, for their immunophenotypic profile^2,3^. Although TC-specific antigenic markers have yet to be discovered, at present CD34 is considered the most reliable one for light microscopic *in situ* identification of TCs, which indeed are also commonly referred to as TCs/CD34^+^ stromal cells^4^. As in the stromal compartment of vascularized organs the expression of CD34 is shared by TCs and endothelial cells of blood vessels, only the simultaneous detection of CD34 and endothelial cell-specific CD31/platelet-endothelial cell adhesion molecule-1 can allow to unequivocally discriminate between CD34^+^CD31^−^ TCs and CD34^+^CD31^+^ vascular endothelial structures, especially when vessels are captured as profiles with no obvious lumen. Other markers including vimentin, platelet-derived growth factor receptor-α or -β, or c-kit/CD117 in combination with CD34 are also regarded as useful for TC identification^2,3^. However, while the expression of CD34 has been definitely demonstrated in stromal cells morphologically identifiable as TCs from a variety of organs, the expression of other markers, such as the above-mentioned, may be weak or inconstant in TCs, possibly being organ/tissue- or animal species-dependent^2,3^.

To date, TCs have been identified in the connective tissue layers of many cavitary organs and in the stromal compartment of non-cavitary organs of humans and various mammals and vertebrate animals^2,3,5–12^, including the skeletal muscle interstitium^10,13–18^. Functionally, TCs are thought to contribute to the maintenance of local tissue homeostasis by participating in many physiological processes^2,3^. In fact, by means of their telopodes TCs connect either with each other *via* homocellular connections, or with different cell types *via* heterocellular connections^19^, thus building a complex three-dimensional interstitial network, possibly serving as a guidance scaffold to define the correct tissue organization and mediate cell-to-cell signaling. Of note, increasing evidence indicates that TCs may be involved in the modulation of the functionality of other tissue resident cells not only in a juxtracrine manner by the establishment of heterocellular junctions, but also in a paracrine one, *via* the release and transfer of extracellular vesicles enriched in many bioactive molecules including mRNAs, microRNAs, long non-coding RNAs, proteins and bioactive lipids to neighboring recipient cells^20–23^.

Focusing on the skeletal muscle tissue, within adult muscles TCs have been found widely distribuited throughout the perimysial and endomysial compartments, where their long-distance spreading telopodes are strategically positioned in the close vicinity of striated myofibers with regenerative features, nerve endings, small blood vessels, and often satellite cells (SCs)^13,15^. Interestingly, extracellular vesicles released by telopodes were also detected near SCs^13^. SCs constitute a small population of mononucleated cells whose name relies on their unique anatomical location at the immediate periphery of skeletal myofibers, just beneath the surrounding basal lamina in intimate association with the myofiber sarcolemma. They are considered the resident adult muscle progenitor/stem cells responsible for the post-natal muscle growth and repair/regeneration^24^. In healthy skeletal muscle tissue, SCs are mitotically quiescent and transcriptionally inactive, accounting for only ~2–10% of the total myonuclei in adult muscles (from 2 × 10^5^ to 1 × 10^6^ cells/g muscle, depending on muscle and myofiber types)^24,25^. In this dormant state SCs express the paired box transcription factor Pax7, which is considered the canonical SC-specific marker necessary for their survival and function^26^, and the myogenic transcription factor Myf5 (detectable in ~90% of quiescent SCs) but not the myogenic regulatory factors, namely MyoD or myogenin^25^. In damaged muscles, the percentage of SCs may increase up to ~10–15%. In response to signals coming mainly from injured myofibers and infiltrating inflammatory cells, indeed, SCs exit from the quiescent state to re-enter cell cycle and closely recapitulate the steps of embryonic and fetal myogenesis program. Upon tissue damage, at first SCs undergo mitotic division to give rise to a progeny of proliferating adult myoblasts expressing Pax7, Myf5 and MyoD. Subsequently, adult myoblasts downregulate Pax7, acquire myogenin and MRF4/Myf6 expression and differentiate into skeletal myocytes that finally either fuse to one another forming nascent syncytial contractile myofibers or fuse with injured myofibers to repair the damage^25^. Moreover, there is evidence that only a small percentage of SCs are “true” stem cells, that is cells capable of self-renewal to ensure the replenishment of the basal pool of resident SCs recruitable in case of muscle re-injury^27^. The recently discovered close spatial relationship of TCs and SCs in the adult skeletal muscle led to the postulation that TCs might communicate with these resident progenitor cells through both juxtacrine and paracrine intercellular signaling, potentially modulating their behavior and influencing their fate to support muscle tissue repair/regeneration^13^. In line with this assumption, a recent paper investigating the presence and distribution of TCs in human fetal skeletal muscle tissue from lower limbs revealed intriguing changes in these cells during the early steps of the fetal myogenic process^28^. In particular, it has been demonstrated that TCs increase in number from 9 to 11.5 weeks of gestation and, by means of their telopodes, form an extensive reticular network in close relationship with primary and secondary myotubes undergoing maturation. Strikingly, afterwards the number of TCs appears to be robustly reduced in fetal skeletal muscle tissues at 12 weeks of gestation, where mature myotubes become evident, suggesting that TCs may specifically be implicated in myotube development/formation during the early maturation steps of muscle tissue morphogenesis^28^. Besides the aforementioned experimental data, the intriguing hypothesis of a morpho-functional interaction between TCs and SCs during muscle repair/regeneration is also based on the increasing evidence that TCs may establish a specialized relationship with putative stem/progenitor cells endowed with regenerative potential in a variety of healthy, injured or pathological tissues/organs^3,10,29–34^. In this context, TCs may help in recruiting, nursing or guiding such progenitors to proliferate, differentiate and integrate into the tissue architecture. Nevertheless, studies on TCs in the skeletal muscle tissue of mammals are still scanty and, at present, there is no direct experimental evidence to definitely support a morpho-functional TC-SC interaction following skeletal muscle damage. Hence, in the present morphological study we sought to explore for the first time the presence and tissue distribution of TCs in a mouse model of skeletal muscle damage, focusing on their interaction with SCs. The reciprocal *in vitro* behavior of TCs and SCs isolated from single living endomysial sheath-covered muscle fibers was also assessed.

## Results

### Morphological and electrophysiological characterization of *ex vivo* eccentric contraction (EC)-induced skeletal muscle damage model

We first verified a successful construction of the *ex vivo* EC-induced extensor digitorum longus (EDL) skeletal muscle damage model by means of both morphological and electrophysiological analyses.

**Figure 1:**
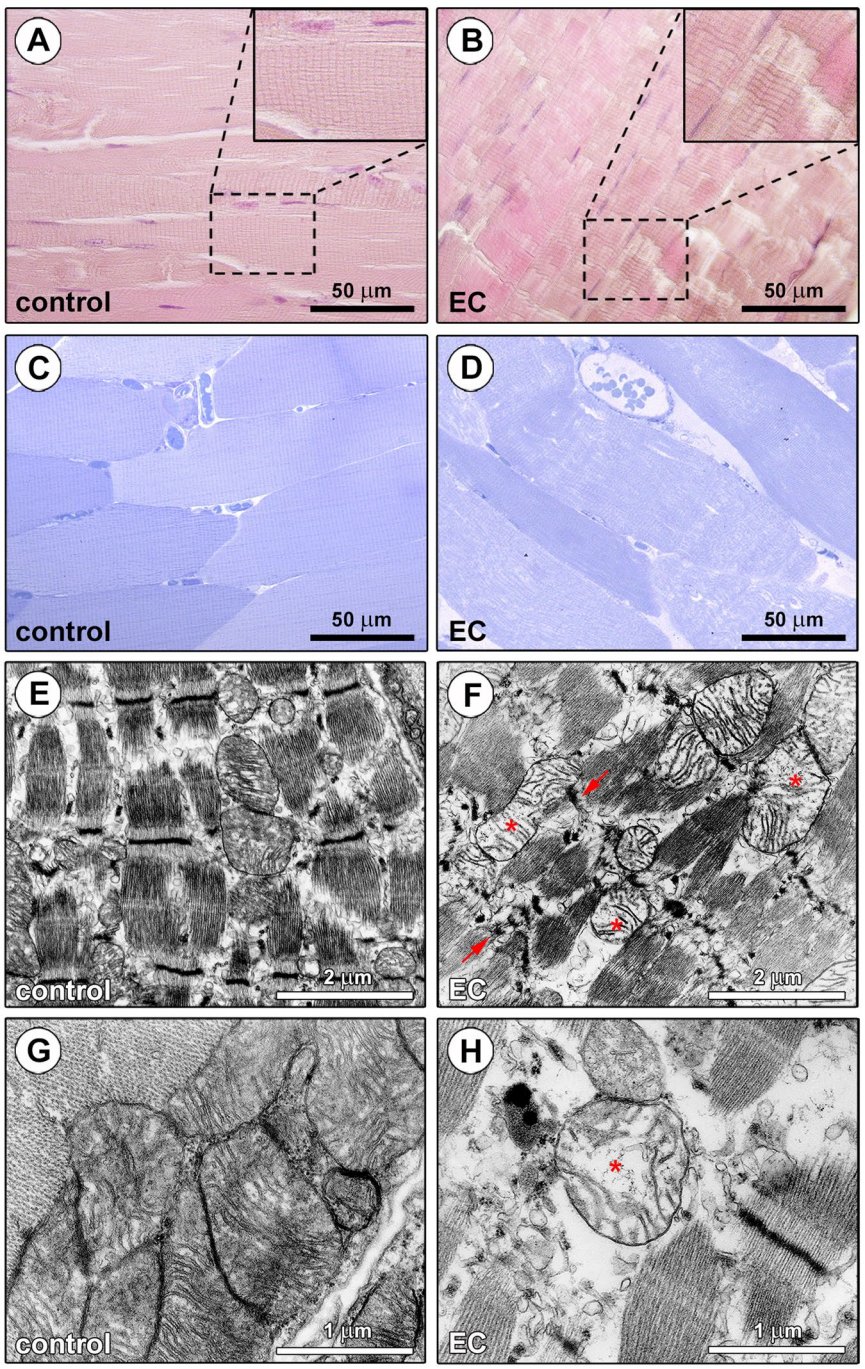
Morphological evaluation of *ex vivo* eccentric contraction (EC)-induced skeletal muscle damage model. **(A,B)** Hematoxylin and eosin staining testifying the normal structure of control muscles and the presence of tissue damages in EC-injured muscles. Higher magnifications of the boxed areas are shown in the insets. Note Z-disc smearing/streaming and focal loss of myofilaments in EC muscles. **(C,D)** Semithin sections of control and EC-injured muscle samples stained with toluidine blue and observed by light microscopy confirming the substantial EC-induced structural abnormalities. **(D)** Swollen and round-shaped myofibers and intramyofiber and interstitial edema are evident in EC-injured samples. **(E-H)** Ultrathin sections of control and EC-damaged muscle samples stained with UranyLess and bismuth subnitrate solutions and examined by TEM. **(F,H)** EC-injured myofibers exhibit an evident disorganization of sarcomere structures and Z-disc disruption (arrows), as well as swollen mitochondria with disarranged or even missing cristae (asterisks). Images are representative of at least 6 sections from each of 5 control and 5 EC-injured muscles.

As displayed in Fig. 1A–D, light microscopic examination of EDL skeletal muscles subjected to EC revealed extensive and severe morphological alterations in comparison with control EDL skeletal muscles. The observation of paraffin-embedded longitudinal sections of control skeletal muscles stained with hematoxylin and eosin confirmed a normal histology, as testified by the presence of long, parallel, and multinucleated myofibers showing minimal size variability, elongated nuclei peripherally located under the sarcolemma and an eosinophilic cross striated sarcoplasm (Fig. 1A). By contrast, longitudinal sections from EC-injured skeletal muscles exhibited myofiber structural damages mainly consisting in Z-disc smearing and streaming and a focal loss of myofilaments (Fig. 1B). These EC-induced morphological alterations were confirmed by the examination of epoxy resin-embedded semithin sections stained with toluidine blue, that also revealed an abnormal morphology of the EC-damaged myofibers (*i.e*. swollen myofibers with rounded shape) and the presence of intracellular and interstitial edema as compared to controls (Fig. 1C,D). At the ultrastructural level, the majority of the EC-injured myofibers displayed severely disarranged myofibrillar sarcomeres and Z-disc disruption, as well as swollen mitochondria with shortened, incomplete or even absent cristae (Fig. 1E–H).

**Figure 2:**
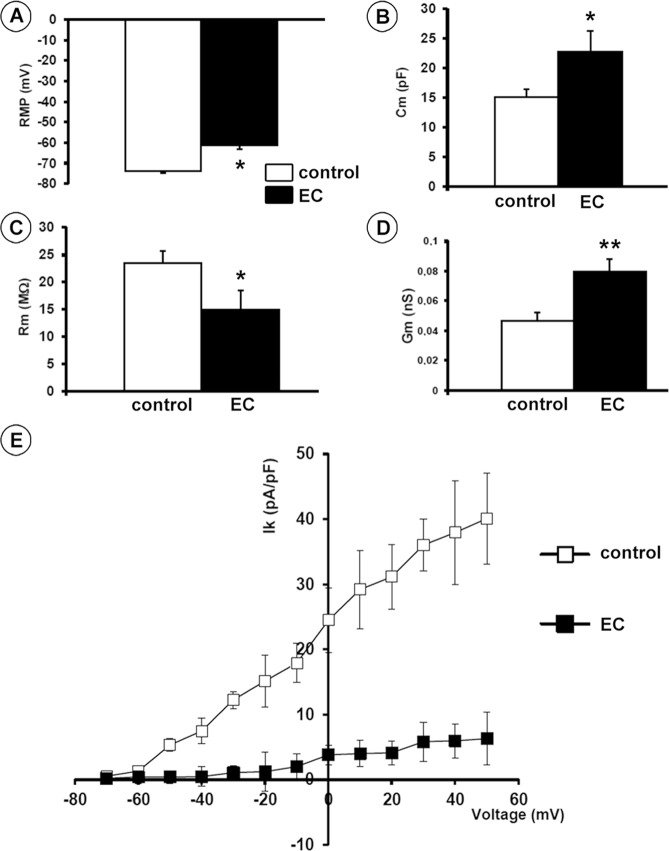
Electrophysiological analysis of the sarcolemnic properties of control and eccentric contraction (EC)-damaged skeletal muscle fibers. **(A)** Resting membrane potential (RMP) recorded in the current clamp mode. **(B–D)** Membrane passive properties of the myofibers measured in voltage-clamp mode: linear capacitance, C_m_, membrane resistance, R_m_, and cell conductance, G_m_. Data are reported as means ± SEM. *P < 0.05, **P < 0.01 *versus* control. **(E)** K^+^ current (I_K_) measured in voltage-clamp mode normalized to cell capacitance C_m_ (pA/pF). The I_K_ values (means ± SEM) recorded in EC muscles, from −50 mV to 60 mV, were statistically different from those recorded in control muscles (*P* < 0.05). All measurements were performed on at least 15 myofibers from each of 5 control and 5 EC-injured muscles.

The electrophysiological analysis performed on single muscle fibers disclosed the occurrence of a number of significant changes in the sarcolemnic functionality of myofibers subjected to EC compared to controls, including (1) resting membrane potential (RMP) depolarization (mean ± SEM values, −61.3 ± 1.08 mV in EC *versus* −74.0 ± 0.57 mV in control myofibers, *P* < 0.05; Fig. 2A); (2) cell capacitance (Cm) increase (mean ± SEM values, 22.8 ± 3.4 pF in EC *versus* 15.1 ± 1.34 pF in control myofibers, *P* < 0.05; Fig. 2B) suggestive of a T-tubular membrane disorganization leading to a sarcolemnic surface increase, consistent with the abnormal myofiber morphology; (3) a reduction in plasma membrane resistance (Rm) values (mean ± SEM values, 14.9 ± 3.5 MΩ in EC *versus* 23.6 ± 2.1 MΩ in control myofibers, *P* < 0.05; Fig. 2C); (4) an increase in conductance (Gm) values (mean ± SEM values, 0.08 ± 0.008 nS in EC *versus* 0.05 ± 0.006 nS in control myofibers, *P* < 0.01; Fig. 2D) suggestive of a more leaky plasma membrane in the damaged myofibers; and (5) a significantly reduced outward K^+^ current (I_K_) amplitude (*P* < 0.05 *versus* control; Fig. 2E) which indicated an alteration of myofiber excitability.

### Morphological evaluation of TCs, SCs and their interaction in *ex vivo* EC-injured skeletal muscles *versus* control muscles

According to relevant recommendations for the morphological study of TCs and considerable reference data^2,3^, the presence and tissue distribution of TCs in EC-injured and control EDL skeletal muscles were investigated either by immunofluorescence (*i.e*. TCs are identifiable as CD34^+^ stromal cells)^2–4^ or by transmission electron microscopy (TEM).

**Figure 3:**
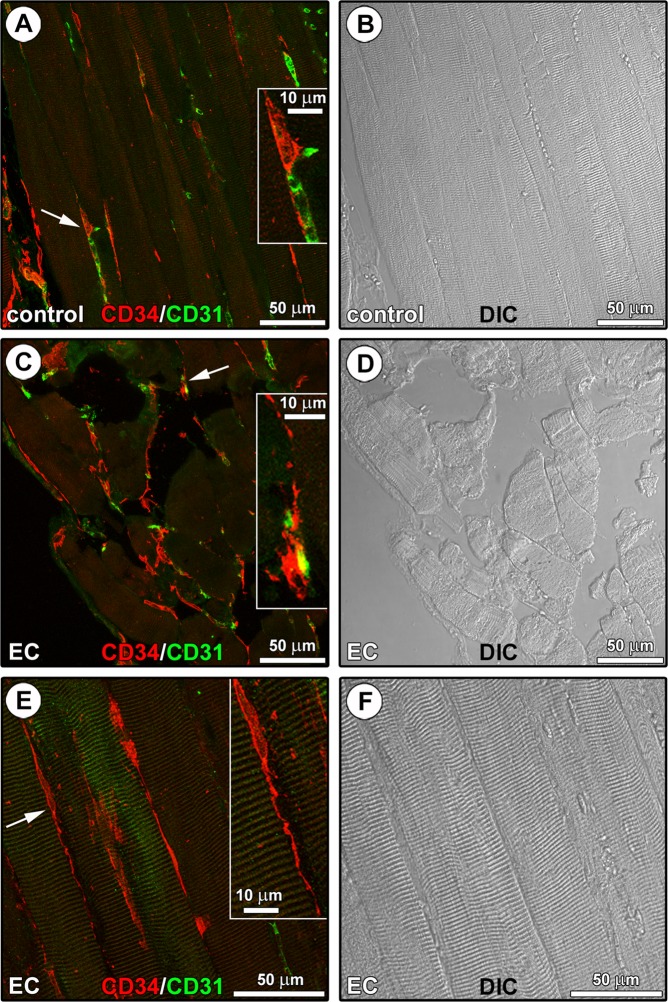
CD34/CD31 confocal immunofluorescence staining of paraffin-embedded tissue sections from control and eccentric contraction (EC)-damaged skeletal muscles. **(A–F)** Representative fluorescence images **(A,C,E)** and differential interference contrast (DIC) images **(B,D,F)** acquired simultaneously to allow a better appreciation of the tissue structure. **(A)** In control muscles, CD34^+^CD31^−^ telocytes (TCs) with distinctive long and thin varicose cytoplasmic prolongations (telopodes) are located in the interstitium alongside the myofibers and in the close vicinity of CD31^+^ vascular structures (arrow and higher magnification in the inset). **(C,E)** An extensive CD34^+^CD31^−^ TC meshwork is evident among myofibers in EC-damaged muscles. Insets: magnifications of the areas indicated by arrows; note the close vicinity of a CD34^+^CD31^−^ TC to a CD34^+^CD31^+^ blood capillary vessel **(C)**, and a CD34^+^CD31^−^ spindle-shaped TC sending a distinctive moniliform telopode over long distance in the interstitial space among two myofibers **(E)**. Images are representative of at least 6 sections from each of 5 control and 5 EC-injured muscles.

As far as confocal laser scanning microscopy is concerned, paraffin-embedded muscle tissue sections underwent CD34/CD31 double confocal immunofluorescence to clearly distinguish TCs, which are characterized by a distinctive CD34^+^CD31^−^ immunophenotype^7,18^, from CD34^+^CD31^+^ vascular endothelial cells (Fig. 3). In the interstitium of both control (Fig. 3A,B) and EC-damaged (Fig. 3C–F) muscles, TCs were identified as CD34^+^CD31^−^ spindle-shaped cells with a relatively small cell body giving rise to very long and thin moniliform/varicose cytoplasmic prolongations (*i.e*. telopodes). In particular, TCs were located alongside the myofibers and in close vicinity of CD31^+^ vascular structures (Fig. 3A,C), and the stromal network formed by their telopodes appeared particularly extended in EC-damaged muscles (Fig. 3C,E).

**Figure 4:**
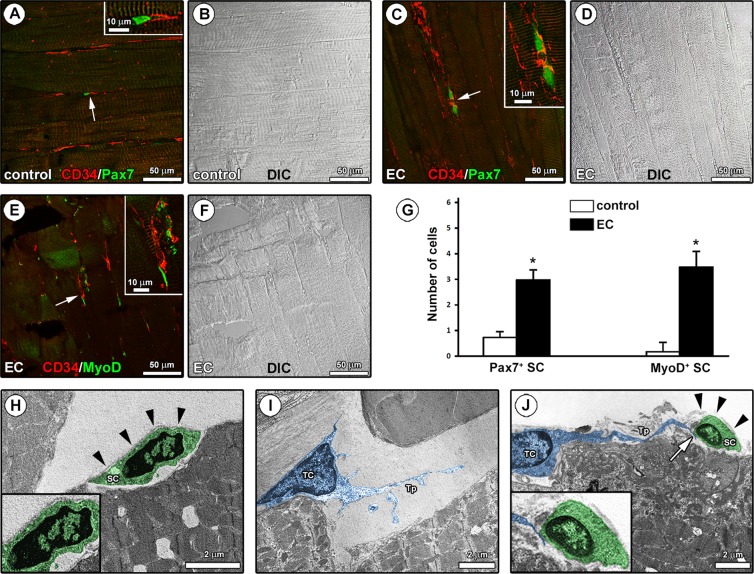
Morphological evaluation of the interaction between telocytes (TCs) and satellite cells (SCs) in control and eccentric contraction (EC)-damaged skeletal muscle samples. **(A–F)** Confocal double immunofluorescence staining of paraffin-embedded muscle sections for **(A,C)** CD34 and Pax7 and **(E)** CD34 and MyoD. Differential interference contrast (DIC) images acquired simultaneously to allow a better visualization of the tissue structure are shown in **(B,D,F)**. **(A,C)** Note the presence of Pax7^+^ SCs at the periphery of the myofibers in the close vicinity of CD34^+^ TCs/telopodes which form a more extended network in EC-damaged muscles with respect to controls (arrows and higher magnifications in the insets). **(E)** In EC-damaged muscles, CD34^+^ TCs/telopodes were observed around activated SCs displaying MyoD nuclear positivity (arrow and higher magnification in the inset). **(G)** Quantitative analysis of the number of Pax7^+^ or MyoD^+^ SCs. Data are means ± SEM. *P < 0.05 *versus* control. **(H–J)** Ultrathin sections of control **(H,I)** and EC-damaged **(J)** muscles stained with UranyLess and bismuth subnitrate solutions and observed by TEM. **(H)** A quiescent SC (digitally colored in green) with a heterochromatic nucleus is located adjacent to the sarcolemma of a skeletal myofiber and completely covered by an intact basal lamina (arrowheads and higher magnification in the inset). **(I)** Note a TC (digitally colored in blue) with a large nucleus surrounded by a scarce cytoplasm and a typical moniliform/varicose telopode (Tp) within the interstitial space in the close vicinity of a skeletal myofiber. **(J)** A close morphological interaction between TCs and activated SCs is found in EC-injured muscles; note a TC (blue) juxtaposed to a damaged myofiber and sending a Tp to contact an activated SC (green) with an euchromatic nucleus and incompletely covered by a fragmented basal lamina (arrowheads and higher magnification in the inset). Images are representative of at least 6 sections from each of 5 control and 5 EC-injured muscles.

Next, in an attempt to evaluate the relationship between TCs and the muscle resident SCs, tissue sections were double immunolabeled to simultaneously detect CD34 and Pax7, the most reliable marker of SCs^26^, or CD34 and MyoD, the SC activation marker^25^ (Fig. 4A–F). As expected, confocal immunofluorescence analysis revealed the presence of few Pax7^+^ SCs at the periphery of myofibers in both control and EC-damaged muscles (Fig. 4A–D). However, two adjacent SCs were often observed in EC-damaged samples, possibly suggesting that these stem cells underwent mitotic division following tissue injury (Fig. 4C). Indeed, quantitative analysis revealed that the number of Pax7^+^ SCs was significantly increased in EC-damaged muscles as compared to controls (*P* < 0.05; Fig. 4G). Of note, a more extended network of CD34^+^ TCs/telopodes surrounding Pax7^+^ SCs was detected in EC-damaged muscles (Fig. 4A–D). Moreover, EC caused a significant increase in MyoD^+^ activated SCs which, indeed, were almost undetectable in control samples (*P* < 0.05; Fig. 4E–G). As shown in Fig. 4E, CD34/MyoD double confocal immunofluorescence clearly highlighted the presence of CD34^+^ TCs/telopodes closely surrounding MyoD^+^ activated SCs.

In keeping with relevant ultrastructural identificative criteria for TCs and SCs^2,35,36^, TEM analysis confirmed the tissue location of both cell types and strengthened the occurrence of peculiar differences in the TC-SC relationship between EC-damaged muscles and control muscles (Fig. 4H–J). In control muscle tissue, SCs were captured in their typical anatomical location, namely at the periphery of myofibers juxtaposed to sarcolemma and completely covered by a basal lamina in the so-called “immediate SC niche” (Fig. 4H). Control muscle SCs appeared to be in a quiescent state, as testified by the heterochromatic appearance of their nucleus (Fig. 4H). Cells ultrastructurally identifiable as TCs were located within the skeletal muscle interstitium, where they typically displayed a spindle-shaped, polygonal or piriform cell body mostly occupied by a large nucleus surrounded by a scarce cytoplasm (Fig. 4I). Telopodes appeared as long and slender cytoplasmic processes abruptly emerging from the cell body and exhibiting a distinctive moniliform morphology conferred by the alternation of podomers (thin segments) and podoms (small dilations) (Fig. 4I). In agreement with the results obtained by CD34 confocal immunofluorescence, TCs/telopodes were usually observed in close relationship with skeletal muscle fibers (Fig. 4I). Notably, in EC-injured muscles TEM analysis revealed the presence of TCs invading the “immediate SC niche”, as demonstrated by the evidence of telopodes passing through a fragmented basal lamina and contacting the underlying activated SCs displaying a swollen appearance and an euchromatic nucleus (Fig. 4J).

### *In vitro* analysis of TCs and SCs from single living endomysial sheath-covered myofibers: differential intercellular interaction after *ex vivo* EC-induced muscle damage

In order to strengthen the aforedescribed tissue findings and to gain further insights into the morphological and potential functional interaction between TCs and SCs, we next performed *in vitro* experiments on skeletal muscle-derived cells. To this purpose, single living myofibers surrounded by their endomysial sheath were collected from control and EC-injured muscles and individually cultured in Matrigel-coated plates.

**Figure 5:**
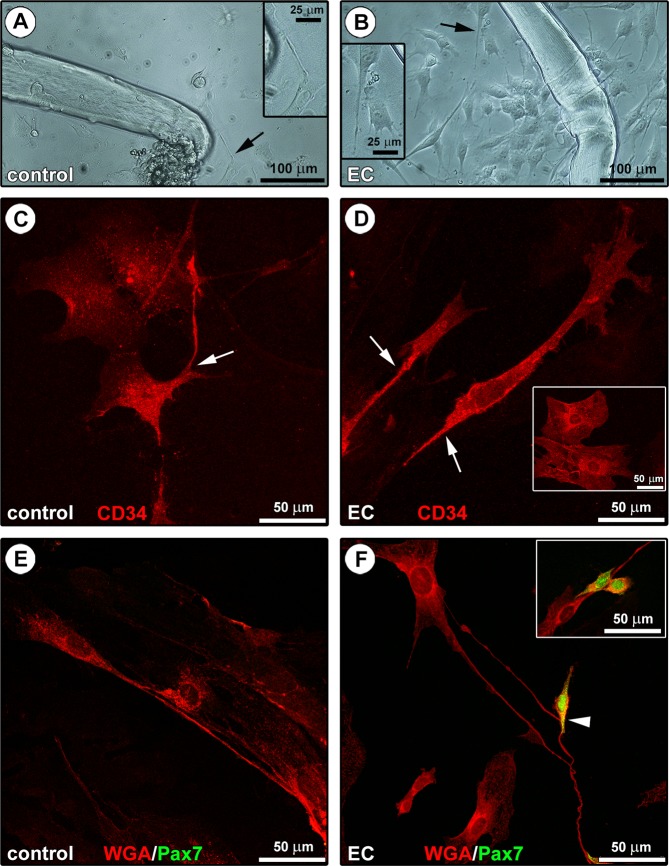
Morphological evaluation of the *in vitro* interaction between telocytes (TCs) and satellite cells (SCs) from single living endomysial sheath-covered myofibers. **(A,B)** Phase-contrast microscopic images of endomysial sheath-covered myofibers from control and eccentric contraction (EC)-damaged muscles after 48 hours of culture on Matrigel-coated plates; sprouting cells appear more numerous around EC-injured myofibers. Note that some of these morphologically different cells display the typical features of TCs (*i.e*. moniliform/varicose telopodes; arrows and higher magnifications in the insets). **(C,D)** CD34 confocal immunofluorescence staining. Cells with a polygonal or spindle-shaped cell body abruptly giving rise to thin moniliform processes (arrows) and displaying a strong CD34 positivity, thus identifiable as TCs, are detected in the heterogeneous cell population derived from either control **(C)** or EC-injured **(D)** myofibers. These cells are distinguishable from typically fried egg-shaped CD34^+^ endothelial cells (**D**, inset). **(E,F)** WGA and Pax7 confocal double fluorescent staining. The presence of telopode-bearing TCs in control and EC-damaged myofiber-derived cells is confirmed by labeling with the membrane dye WGA. Pax7^+^ SCs, undetectable at this experimental time in the control myofiber-derived cell population **(E)**, are instead detected in cell cultures from EC-damaged myofibers **(F)**. Of note, SCs are often observed in cell pairs presumably resulting from mitotic division (**F**, inset) and in contact with TCs/telopodes (**F**, arrowhead and inset). Images are representative of at least 6 cell preparations from each of 3 control and 3 EC-injured muscle samples.

After 48 hours of culture, phase-contrast microscopy analysis revealed a heterogeneous cell population surrounding both control and EC-injured myofibers, though these cells were much more numerous in EC samples (Fig. 5A,B). Such heterogeneous population included cells with the typical morphological and immunophenotypical features of TCs (Fig. 5A–D). Indeed, TCs were identified in culture as cells exhibiting long and thin moniliform/varicose processes abruptly emerging from a polygonal or spindle-shaped cell body (Fig. 5A,B), as well as a strong positivity for the CD34 antigen as judged by confocal immunofluorescence analysis of fixed cells (Fig. 5C,D). Moreover, thanks to the presence of their characteristic prolongations (*i.e*. telopodes), the immunostained TCs were clearly distinguishable from fried egg-shaped endothelial cells that also expressed CD34 (Fig. 5C,D). The morphological features of the telopode-bearing TCs were confirmed also by confocal fluorescence analysis of cells derived from control and EC-damaged myofibers labeled with the membrane dye wheat germ agglutinin (WGA) (Fig. 5E,F). Of note, in agreement with the results obtained by confocal immunofluorescence and TEM analyses of muscle tissue samples, the telopodes of TCs were often observed in contact with the Pax7^+^ SCs sprouted from EC-damaged myofibers (Fig. 5F).

**Figure 6:**
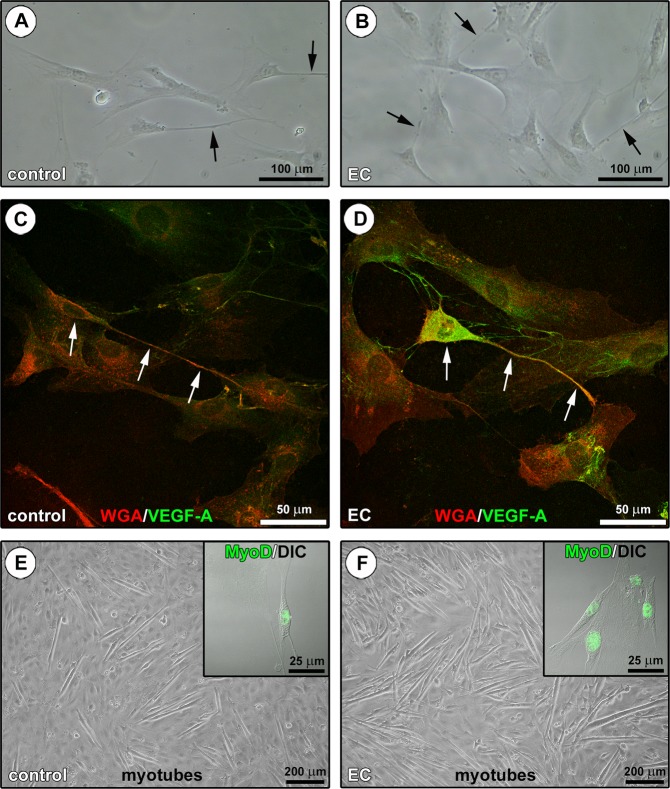
Assessment of the *in vitro* behavior of stromal cell-enriched or satellite cell (SC)-enriched populations derived from control and eccentric contraction (EC)-damaged endomysial sheath-covered myofibers. **(A,B)** Phase-contrast microscopic images of the stromal cell-enriched population cultured after myofiber removal. Note the presence of cells identifiable as telocytes (TCs) with long moniliform/varicose cytoplasmic process (arrows). **(C,D)** WGA and vascular endothelial growth factor (VEGF)-A confocal double fluorescent staining of the stromal cell-enriched population. Cells morphologically identifiable as TCs (arrows) from control **(C)** and EC-damaged **(D)** myofibers appear almost negative and strongly positive for VEGF-A, respectively. In the EC-damaged myofiber-derived cell population, note a TC displaying strong VEGF-A immunoreactivity and contacting with its cytoplasmic processes multiple neighboring cells **(D)**. **(E,F)** Phase-contrast microscopic images showing the formation of myotubes in the SC-enriched population after 5 days of culture in gelatin-coated plates. Note that myotubes are more numerous in the SC-enriched culture from EC-damaged myofibers compared to controls. Insets: superimposed differential interference contrast (DIC) and confocal immunofluorescence images of SCs cultured for 48 hours and immunostained for MyoD. Consistent with their greater tendency to fuse into myotubes, more numerous SCs sprouted from EC-damaged myofibers exhibit MyoD^+^ nuclei compared with controls. Images are representative of at least 6 cell preparations from each of 3 control and 3 EC-injured muscle samples.

In order to further explore the potential functional significance of the interaction between TCs and SCs, we investigated the expression of vascular endothelial growth factor (VEGF)-A in a stromal cell-enriched population obtained after myofiber removal (Fig. 6A,B) because (1) VEGF-A has been proved to promote myoblast proliferation/differentiation^37–43^, and (2) TCs have been reported to highly express and secrete this growth factor^13–15,20–23,44^. As shown by confocal fluorescence analysis of cells stained with WGA and immunostained for VEGF-A, cultured stromal cells with TC morphological traits from EC-damaged myofibers expressed high levels of VEGF-A, that instead was almost undetectable in TCs from controls (Fig. 6C,D). The parallel analysis of the activation and differentiation of a SC-enriched population derived from the same samples clearly revealed a greater number of MyoD-expressing cells sprouted from EC-damaged myofibers, as well as a greater tendency of these cells to fuse into myotubes after 5 days of confluent culture in proliferation medium in comparison to controls (Fig. 6E,F).

## Discussion

It has been clearly proven that adult skeletal muscle tissue possesses a remarkable ability to regenerate in response to focal injuries. Muscle regeneration represents a highly coordinated multi-step process, starting from degeneration and/or necrosis of injured myofibers and ending with formation of new contractile myofibers, aimed to the morpho-functional restoration of damaged muscle tissue^45,46^. Although considerable work has demonstrated that many different cell types residing within the muscle tissue or recruited *via* the bloodstream may contribute to new myofiber formation owing to their inducible myogenic potential (the so-called myogenic non-SCs), muscle resident SCs are broadly regarded as the main players in such a regenerative process^24,47^.

The behavior and fate specification of SCs are profoundly influenced by the dynamic interplay they establish with the components of the surrounding microenvironment. In healthy intact muscles, the local microenvironment essentially acts for maintaining SCs in a quiescent state; by contrast, during physiological tissue repair after an injury, it releases biochemical and physical signals which promote the activation of SCs and their proper progression into the myogenic differentiation program as well as SC self-renewal^27,48,49^. Specifically, the whole microenvironment surrounding SCs may be subdivided into the so-called “immediate SC niche” and the “microenvironment beyond the immediate niche”. The “immediate SC niche” is represented by the specific anatomical location where SCs reside (*i.e*. between the basal lamina and the sarcolemma of terminally differentiated skeletal myofibers) and includes different extracellular matrix (ECM) components, signaling molecules diffusing between the myofiber and the SC, cell surface receptors mediating both cell-to-ECM and cell-to-cell interactions or binding regulatory factors, as well as receptors located in the basal lamina sequestering inactive growth factor precursors which serve as a local reservoir to be rapidly activated after damage^24,47,50,51^. The “microenvironment beyond the immediate niche”, instead, comprises both the local milieu consisting in different interstitial cells and the vascular and neural networks with their associated secretable factors within the stroma between the myofibers of a muscle fascicle, and the systemic milieu including several molecular and cellular signals coming from the entire muscle belly along with neighboring skeletal muscles and bones^46,48,50^. In particular, the juxtacrine and paracrine mutual interactions that SCs establish with the tissue resident or recruited interstitial cells appear crucial in the regulation of the SC quiescent state and even more activation, proliferation and differentiation after injury. In fact, as compared to intact muscles, following tissue damage a high number of cells, putatively supporting SC-mediated regeneration, has been found in the activated microenvironment in the close vicinity of SCs^46,49,52^. These interstitial cells mainly include pro-inflammatory (M1) and anti-inflammatory/pro-regenerative (M2) macrophages^36,53,54^, fibroblasts^55^, mesenchymal stem/stromal cells such as fibro-adipogenic progenitors^56^, capillary endothelial cells and perycites^38,57,58^.

Only recently, TCs entered this scenario as a new distinctive type of interstitial cells that may potentially behave as nursing cells for SC-mediated skeletal muscle regeneration. However, it must be frankly recognized that such supporting role for TCs has only been supposed on the basis of their *in situ* identification in the close vicinity of SCs in healthy skeletal muscles^10,13–15,17^, their possible role during early myogenesis^28^, and their well-documented interaction and/or cross-talk with stem/progenitor cells in other organs^3,10,29–34^. Indeed, at present, the effective occurrence of a morpho-functional interplay between TCs and SCs in injured skeletal muscles has yet to be demonstrated with certainty. Hence, we considered that experimental studies aimed at investigating the reciprocal behavior of TCs and SCs in an injured muscle are essential to clarify whether TCs may take part in the regenerative events following muscle tissue damage and, in particular, whether muscle resident SCs and TCs may be privileged partners in such context. In the present study we have described for the first time the distribution of TCs in murine skeletal muscles damaged by forced EC and provided novel experimental evidence supporting their morpho-functional interaction with activated SCs. In particular, confocal immunofluorescence analysis of intact control muscle sections revealed the presence of CD34^+^CD31^−^ elongated, thin and varicose processes organized in an interstitial network alongside the striated myofibers and around blood vessels, consistent with the previous description of telopodes of TCs in the endomysial and perimysial sheaths of skeletal muscle^10,13–15,17^. Notably, in EC-damaged muscles this CD34^+^CD31^−^ telopode network appeared particularly extended and was preferentially arranged around activated SCs that displayed Pax7 and MyoD nuclear positivity and were located in their distinctive site at the periphery of striated myofibers. As expected, and in agreement with a previous report describing the same *ex vivo* model of muscle damage^59^, the Pax7^+^ SCs were more numerous in EC-damaged muscles as compared with controls indicating that these cells presumably left their quiescent state to re-enter cell cycle. In fact, it is noteworthy that SCs positive for MyoD, the myogenic regulatory factor regarded as an early marker of cell activation and myogenic commitment^25^, were detected in damaged muscles, but not in the intact controls, and were captured in a close spatial relationship with TCs/telopodes. Furthermore, TEM analysis clearly highlighted the close heterocellular interaction between TCs and activated SCs in EC-damaged muscles. Indeed, in these samples, interstitial cells located among striated myofibers and fulfilling the TC ultrastructural identificative criteria were seen to invade the “immediate SC niche”, where, by means of their telopodes, crossed the fragmented basal lamina and contacted the underlying SCs which showed signs of activation, namely cell swelling and lower chromatin density, and appeared incompletely covered by the basal lamina^35,36^. In this regard, it is conceivable that a fragmented basal lamina results functional to the prompt migration of activated SCs toward the injury site^35,36,51^, and that activated SCs may contribute themselves to the break down of the basal lamina. In fact, previous studies have shown the capability of SCs to express and secrete functional proteolytic enzymes selectively digesting individual components of the ECM (*e.g*. matrix metalloproteinase-2 and -9) and their specific tissue inhibitors^51,59–62^. A positive correlation between the expression and activity of matrix metalloproteinases in myoblastic cells *(i.e*. C2C12 myoblasts and primary murine SCs) and their migratory ability across type-I collagen substrate or basal lamina was also demonstrated^62^. When considering that TCs have also been reported to express matrix metalloproteinases^63^, it is tempting to speculate that they might either cooperate with SCs in the digestion of the basal lamina or even mediate the release of tethered pro-myogenic factors from ECM thus contributing to SC activation^51,64^. Besides *in situ* tissue analyses, in the present work the preferential interaction of TCs and SCs from EC-damaged muscles was also confirmed *in vitro* in a set of experiments performed by culturing single skeletal myofibers surrounded by their endomysial sheath, and the sprouting TCs and SCs. A further intriguing result of our study is the increase in VEGF-A expression found in cultured TCs from EC-damaged muscles. This observation is consistent with previous reports demonstrating an ability of TCs from different tissues, including skeletal muscle, to produce VEGF whose secretion might occur *via* the release of extracellular vesicles^13–15,20–23,44^, and sheds some light on the potential paracrine mechanisms by which TCs may stimulate SC activation and foster myogenic differentiation. In support, it is interesting to note that a prominent role of VEGF-A in modulating the myoblast/SC behavior is being increasingly reported in different healthy and pathological conditions^37–43^. Thus, the potential functional role of TC-derived VEGF-A in mediating TC-SC interactions is worth of further investigation. In addition, we believe that future studies aimed at evaluating the possible involvement of juxtacrine mechanisms in mediating the cross-talk between TCs and SCs deserve a particular attention.

In conclusion, although we recognize some limitations of our study (*i.e*. (1) the *ex vivo* model of muscle damage does not allow to recapitulate all the phases of muscle repair/regeneration process, and (2) the *in vitro* experimentation obviously eliminates many paracrine/juxtacrine mechanisms that may regulate *in situ* intercellular interactions and cell functionality), the present findings contribute either to extend our knowledge of TC biology or to shed additional light on the cellular and molecular mechanisms regulating SC functionality, establishing for the first time that SCs and TCs may represent close neighbors and privileged functional partners in a damaged skeletal muscle condition. This study may therefore be considered as a basal groundwork for further investigations devoted to comprehensively decipher the molecular mechanisms underlying the TC-SC interplay during skeletal muscle tissue repair and potentially representing new attractive targets in the field of regenerative medicine.

## Methods

### Murine model of *ex vivo* EC-induced skeletal muscle injury

Young adult male Swiss mice (25–30 g) were gradually anesthetized with Xylazine (5 mg/kg; Sigma-Aldrich, St. Louis, MO, USA), Lidocaine (2% w/v; Sigma-Aldrich) and Zoletil (Virbac S.r.l., Milan, Italy) from a low dose (20 mg/kg) to a higher dose (40 mg/kg) to avoid a respiratory arrest. The anesthetic mixture was administered intramuscularly. An adequate depth of anesthesia was maintained so that the mice were unresponsive to tail pinch/tactile stimulation. EDL muscles were carefully excised from each limb of the animal: one muscle was subjected to forced EC in isometric condition as previously reported^59^ while the contralateral, not injured by EC, was used as an internal control. In particular, muscle injury was achieved through stretching (stretch extent ~20% of the muscle resting length) and exposure to a series of contractures (ten cycles of 1 minute each) in a solution containing a high K^+^ concentration (mM): 150 K-glutamate, 2.0 MgCl_2_, 10 KOH, 10 TES e 1.0 K_2_-EGTA (Sigma-Aldrich). After each cycle of EC, muscles were recovered in physiological Ringer-Krebs solution containing (mM) 140 NaCl, 5 KCl, 2.5 CaCl_2_, 1 MgCl_2_, 10 D-glucose and 10 HEPES (Sigma-Aldrich) for 4 minutes. After EDL muscle excision, anesthetized mice were sacrificed by cervical dislocation. All efforts were made to minimize animal suffering and to reduce the number of animals used. A total of 18 mice were used in the experimental procedures. Animals were housed in a Laboratory Animal Facility (CeSAL, Centro Stabulazione Animali da Laboratorio, University of Florence), fed with standard laboratory diet and tap water *ad libitum*, kept at 23 ± 1 °C with a 12 hour light/dark cycle. Animal manipulations were carried out in accordance with the Directive 2010/63/EU of the European Parliament and of the European Union council (22 September 2010) on the protection of animals used for scientific purposes. The study protocol was approved by the Institutional Animal Care and Use Committee of the University of Florence. The ethical policy of the University of Florence complies with the Guide for the Care and Use of Laboratory Animals of the US National Institutes of Health (NIH Publication No. 85–23, revised 1996; University of Florence assurance number: A5278-01).

### Histochemistry

Both EC-injured and control EDL muscles (n = 5 each) were fixed with 10% formalin in PBS, dehydrated with a graded alcohol series, cleared in xylene, and embedded in paraffin. Muscles sections (5 μm thick) were deparaffinized and routinely stained with hematoxylin and eosin. Tissue morphology was then observed under a light microscope (Leica DM4000 B) equipped with a DFC310 FX 1.4-megapixel digital color camera and the software application suite LAS V3.8 (Leica Microsystems, Mannheim, Germany).

### TEM

Samples were processed for ultrastructural analysis by TEM as previously reported with minor modifications^59^. Briefly, EC-injured and control EDL muscle specimens (n = 5 each) were fixed with 4% cacodylate-buffered glutaraldehyde (pH 7.4) solution for 2 hours, post fixed in 1% OsO_4_ in 0.1 M phosphate buffer (pH 7.4) for 1 hour at room temperature, dehydrated in a graded acetone series, passed through propylene oxide and embedded in Epon 812 (Sigma-Aldrich). Semithin sections (2 μm thick) were stained with toluidine blue. Ultrathin sections (60 nm thick) were contrasted with UranyLess stain (Electron Microscopy Sciences, Foster City, CA, USA) and alkaline bismuth subnitrate, and then examined using a Jeol 1010 electron microscope (Jeol, Tokyo, Japan) at 80 kV. TCs and SCs detected in electron microscopy images were digitally colored in blue and green, respectively, using Adobe Photoshop CS6 software (Adobe Systems, San Jose, CA, USA).

### Electrophysiological analyses

Electrophysiological analyses were conducted on EC-injured and control EDL muscles (n = 5 each) essentially as previously reported^59,65–67^. The muscle was continuously superfused during recordings at a rate of 1.8 ml/minute (Pump 33, Harvard Apparatus LTD, Edenbridge, Kent, UK) in the experimental chamber with the Ringer-Krebs solution (mM): 120 NaCl, 5 KCl, 2 CaCl_2_, 1 MgCl_2_, 5.5 HEPES, and 1 D-glucose (Sigma-Aldrich), at room temperature (22 °C). RMP was recorded in the current clamp mode by inserting a microelectrode into a single skeletal muscle fiber of isolated EC-injured and control EDL muscles (at least 15 myofibers for each muscle). The microelectrodes were pulled by a micropipette vertical puller (Narishige PC-10; Narishige International Inc., East Meadow, NY, USA) from borosilicate glass (GC 100–7.5; Harvard Apparatus LTD) and were filled with an internal filling pipette solution containing (mM): 130 KCl, 10 NaH_2_PO_4_, 0.2 CaCl_2_, 1 EGTA, 5 MgATP and 10 HEPES/KOH (pH 7.2) (Sigma-Aldrich). The tip resistance was about 60 MΩ. The junction potential of the electrode was measured prior to make the patch, and then it was subtracted from the recorded membrane potential. The RMP was recorded in physiological solution by using a stimulus waveform I = 0 pA. The membrane passive properties of the fibers (linear capacitance, C_m_, membrane resistance, R_m_, and cell conductance, G_m_) as well as K^+^ current (I_K_) were measured in voltage-clamp mode. To estimate the membrane passive properties two 75-ms long voltage step pulses to −80 and −60 mV were usually applied, starting from a holding potential (HP) of −70 mV. To evoke the ionic transmembrane currents, we applied 1-s long voltage pulses ranging from −80 to 50 mV in 10-mV increments (HP = −80 mV). The P/4 procedure allowed us to cancel on line any leakage and voltage-independent ionic currents from the records. The current amplitude (I) was normalized to cell capacitance C_m_ (in pA/pF) to properly compare the currents recorded from different fibers. The I/C_m_ values represent the current density (in pA/pF). The Digital-to-analog and analog-to-digital conversions were carried out by using a Digidata 1200 interface (Axon Instruments, Burlingame, CA, USA). pCLAMP programs (version 6.02 and 9.0, Axon Instruments) were used for stimulation, recordings and data acquisition. The condition of the fibers was constantly checked by monitoring the RMP and R_m_. Fibers remained stable for about 150–240 minutes.

### *In vitro* culture of SCs and stromal cells from single living endomysial sheath-covered myofibers

Single endomysial sheath-covered living myofibers were isolated from both EC-injured and control EDL muscles (n = 3 each; 24 myofibers for each muscle) digested in a DMEM solution containing 0.2% collagenase type I (Sigma-Aldrich) and processed according to previously published protocols^59,62^. Briefly, single living myofibers were isolated from the digested muscle by a gentle mechanical trituration using a Pasteur pipette, and individually transferred in a 24-well Matrigel-treated plate (BD Biosciences, San Jose, CA, USA) or glass coverslips and cultured for 48 hours in SC proliferation medium containing DMEM plus 20% fetal bovine serum (FBS; Sigma-Aldrich), 10% horse serum (Sigma-Aldrich), 0.5% chicken embryo extract (Sera Laboratories International Ltd, Horsted Keynes, UK) and 100 U/ml penicillin-streptomycin (Sigma-Aldrich) to allow cell sprouting. Then, the myofibers were removed and the derived cells were cultured in proliferation medium until reaching 80% of confluence. The cells were then detached using a solution of 0.05% trypsin–0.03% ethylenediaminetetraacetic acid (Sigma-Aldrich) for 5 minutes at 37 °C and re-seeded in a culture plate for 20 minutes with fresh proliferation medium. After 20 minutes, the non-adherent cells (mostly SCs) were collected and cultured (on gelatin-coated culture plates or glass coverslips) in SC proliferation medium, while the adherent ones (mostly stromal cells, including TCs)^62^ were washed and cultured (on culture plates or glass coverslips) in DMEM plus 20% FBS and 1% penicillin/streptomycin at 37 °C in a humidified atmosphere of 5% CO_2_. The cells were monitored and observed every day under an inverted phase-contrast microscope (Nikon Diaphot 300, Nikon, Tokyo, Japan). Myotube formation from confluent EC and control myofiber-derived SCs cultured for 5 days in their specific proliferation medium was observed under an inverted phase-contrast microscope (Nikon Diaphot 300, Nikon).

### Confocal immunofluorescence

Sections (5 µm thick) of paraffin-embedded EC-injured and control EDL muscle tissue samples were deparaffinized, rehydrated, permeabilized with cold acetone for 10 minutes and then incubated with a solution containing 10% glycine in PBS for 10 minutes to quench autofluorescence. Muscle sections were blocked with 0.5% bovine serum albumin (BSA; Sigma-Aldrich) and 0.2% gelatin in PBS for 30 minutes and incubated at 4 °C overnight with the following primary antibodies: rabbit polyclonal anti-CD34 (1:50; Abcam, Cambridge, UK), rat polyclonal anti-CD31 (1:100; Abcam), mouse monoclonal anti-Pax7 (1:100; Santa Cruz Biotechnology, Santa Cruz, CA, USA), mouse monoclonal anti-MyoD (1:100; Santa Cruz Biotechnology). Stromal cell/TC-enriched and SC-enriched culture grown on glass coverslips were fixed with 0.5% paraformaldehyde in PBS for 10 minutes at room temperature, permeabilized with cold acetone for 3 minutes and incubated with a blocking solution containing 0.5% BSA (Sigma-Aldrich) and 0.2% gelatin in PBS for 20 minutes. Cell preparations were then incubated overnight at 4 °C with the following antibodies: rabbit polyclonal anti-CD34 (1:50; Abcam), mouse monoclonal anti-Pax7 (1:100; Santa Cruz Biotechnology), mouse monoclonal anti-MyoD (1:100; Santa Cruz Biotechnology) or mouse monoclonal anti-VEGF-A (1:80; Santa Cruz Biotechnology). In some experiments, cell plasma membrane was stained with WGA Tetramethylrhodamine conjugate (1:250; Thermo Fisher Scientific, Waltham, MA, USA) for 15 minutes at room temperature in the dark. Primary antibodies were revealed using specific anti-rabbit/anti-mouse/anti-rat Alexa Fluor 488-conjugated IgG (1:200; Molecular Probes, Eugene, OR, USA) or 568-conjugated IgG (1:100; Molecular Probes) for 1 hour at room temperature. Negative controls were performed by replacing primary antibodies with non-immune serum, while cross reactivity of secondary antibodies was verified by omitting primary antibodies. Immunolabeled samples were rinsed in PBS, mounted using an antifade gel mounting medium (Biomeda Gel mount; Electron Microscopy Sciences) and examined with a Leica TCS SP5 confocal microscope (Leica Microsystems) equipped with a HeNe/Ar laser source, a Leica Plan Apo 63×/1.43NA oil immersion objective, for fluorescence measurements and differential interference contrast (DIC) optics. Series of optical sections (1024 × 1024 pixels each; pixel size 204.3 nm) 0.4 µm in thickness were taken throughout the depth of the samples at intervals of 0.4 µm and the images projected onto a single ‘extended focus’ image. DIC images were captured to visualize the precise tissue structure and localization of the immunostaining. Counts of Pax7^+^ and MyoD^+^ SCs were performed on 5 random 200 μm^2^ optical square fields (63×) containing at least 10 muscle fibers, under the confocal Leica TCS SP5 microscope, in each tissue section. At least three tissue sections from each EDL muscle (n = 5) were analyzed. Counting was performed by two independent observers and the count numbers were then averaged.

### Statistical analysis

Data are reported as means ± SEM. We used unpaired Student’s *t*-test for comparisons between data obtained from EC-damaged and control muscles. *P* < 0.05 was considered statistically significant.

## Data Availability

All relevant data are within the paper.
